# Quality of life in restorative *versus* non-restorative resections for rectal cancer: systematic review

**DOI:** 10.1093/bjsopen/zrab101

**Published:** 2022-01-18

**Authors:** Samuel Lawday, Nicholas Flamey, George E Fowler, Matthew Leaning, Nadine Dyar, Ian R Daniels, Neil J Smart, Christopher Hyde

**Affiliations:** HeSRU, Royal Devon and Exeter Hospital, Exeter, UK; Bristol Centre for Surgical Research, University of Bristol, Bristol, UK; College of Medicine and Health, University of Exeter, Exeter, UK; HeSRU, Royal Devon and Exeter Hospital, Exeter, UK; HeSRU, Royal Devon and Exeter Hospital, Exeter, UK; Bristol Centre for Surgical Research, University of Bristol, Bristol, UK; HeSRU, Royal Devon and Exeter Hospital, Exeter, UK; HeSRU, Royal Devon and Exeter Hospital, Exeter, UK; HeSRU, Royal Devon and Exeter Hospital, Exeter, UK; HeSRU, Royal Devon and Exeter Hospital, Exeter, UK; College of Medicine and Health, University of Exeter, Exeter, UK; College of Medicine and Health, University of Exeter, Exeter, UK

## Abstract

**Background:**

Low rectal cancers could be treated using restorative (anterior resection, AR) or non-restorative procedures with an end/permanent stoma (Hartmann’s, HE; or abdominoperineal excision, APE). Although the surgical choice is determined by tumour and patient factors, quality of life (QoL) will also influence the patient's future beyond cancer. This systematic review of the literature compared postoperative QoL between the restorative and non-restorative techniques using validated measurement tools.

**Methods:**

The review was registered on PROSPERO (CRD42020131492). Embase and MEDLINE, along with grey literature and trials websites, were searched comprehensively for papers published since 2012. Inclusion criteria were original research in an adult population with rectal cancer that reported QoL using a validated tool, including the European Organization for Research and Treatment of Cancer QLQ-CR30, QLQ-CR29, and QLQ-CR38. Studies were included if they compared AR with APE (or HE), independent of study design. Risk of bias was assessed using the Risk Of Bias In Non-Randomized Studies of Interventions (ROBINS-I) tool. Outcomes of interest were: QoL, pain, gastrointestinal (GI) symptoms (stool frequency, flatulence, diarrhoea and constipation), and body image.

**Results:**

Nineteen studies met the inclusion criteria with a total of 6453 patients; all papers were observational and just four included preoperative evaluations. There was no identifiable difference in global QoL and pain between the two surgical techniques. Reported results regarding GI symptoms and body image documented similar findings. The ROBINS-I tool highlighted a significant risk of bias across the studies.

**Conclusion:**

Currently, it is not possible to draw a firm conclusion on postoperative QoL, pain, GI symptoms, and body image following restorative or non-restorative surgery. The included studies were generally of poor quality, lacked preoperative evaluations, and showed considerable bias in the data.

## Teaser

This systematic review compared quality-of-life outcomes for patients who had undergone restorative and non-restorative resections for low rectal cancer. It identified low-quality papers with a high risk of bias addressing this clinically important question. No different in global quality of life was identified, but a difference in patient symptoms was noted.

## Introduction

The treatment for rectal cancer has changed significantly over the past 20 years with the introduction of MRI-based, multidisciplinary team-directed, individualized patient care and the selective use of neoadjuvant therapies^1^. For most patients with rectal cancer, surgery continues to be mainstay of curative treatment. Surgical techniques are based on total mesorectal excision (TME), and comprise either a restorative rectal resection (anterior resection, AR) with anastomosis or an excisional rectal technique with an end/permanent stoma (Hartmann’s, HE; or abdominoperineal excision, APE)^2^. Over the past 100 years, debate has existed regarding which surgical technique (restorative or excisional) provides the best outcome for the patient, with trends towards restorative surgery^3^. It is recognized that each operative approach is different and both techniques are not suitable for all patients^4^. Tumour stage, morphology, and clinical presentation all influence patient outcomes, and identifying the true impact of individual operations can be difficult. Clinical research, systematic reviews, and meta-analyses of rectal cancer outcomes have concentrated on the technical elements and technology used to perform the procedure. These include reviews on robotic *versus* laparoscopic^5^^,^^6^, open *versus* laparoscopic^7^^,^^8^, and transanal TME ^9^^,^^10^ surgery, most of which focused on demonstrating surgical and oncological equivalence or cost–benefit of the procedural approach. There has been little focus on comparing quality of life (QoL) or patient-reported outcome measures (PROMs).

A number of tools, such as EQ-5D™ (EuroQol Group, Rotterdam, the Netherlands), Short Form 36, Functional Assessment of Cancer Therapy—Colorectal (FACT-C), and European Organization for Research and Treatment of Cancer (EORTC) QLQ-CR29, QLQ-CR30, and QLQ-CR38, have all been validated to accurately reflect changes in patients’ QoL^11–15^. Some of these tools are generic and global; EQ-5D™ measures overall health status and is applicable in any condition. EORTC QLQ-CR29 and QLQ-CR30 questionnaires are global QoL tools specific for colorectal cancer. These tools have been shown offer validity and reliability in population groups to ensure that the results reflect true patient experience. A paucity of high-quality comparative PROM and QoL data following rectal cancer surgery to aid in patients’ decision-making between surgical options was reported in 2012^16^. Comparison of these two surgical techniques is challenging, and there is currently no one PROM that can aid this. Indeed, social interaction, body image, and overall QoL influence the patient’s future in living with and beyond cancer^17^ and, when obtaining informed consent, patients should be advised objectively about the treatment options available^18^. This systematic review of the literature on low rectal cancer compared restorative with non-restorative resection, focusing on validated QoL measures.

## Methods

This systematic review was registered on PROSPERO (CRD42020131492), and complies with PRISMA and AMSTAR guidelines^19^^,^^20^. Using a PICO search, the population of interest comprised patients with low rectal cancer undergoing an intervention of AR or restorative bowel resection compared with those who had APE or non-restorative bowel resection, with an outcome measured using a validated QoL tool. The review question was established *a priori*, with inclusion and exclusion criteria, and the risk-of-bias tool chosen before completion of the search. A comprehensive search of Embase and MEDLINE was completed. The search strategy is available in supplementary material (*Appendix S1*). This built on the published Cochrane systematic review^16^, using only papers published from this date. References of included articles were screened for suitable papers. Grey literature was searched in the British Library Thesis repository and Grey Literature search engine. ISRCTN and ClinicalTrials.gov were screened for suitable trials. The search was completed on 20 June 2020. Published data comparing validated QoL outcomes in adults undergoing radical surgery for rectal cancer were included. Transabdominal surgical techniques with curative intent were included. There was no limit based on follow-up time. Randomized and observational studies were included, although randomization between the two groups was thought to be unlikely.

Inclusion criteria were: surgical intervention for rectal cancer in adults aged over 18 years; surgery performed with curative intent; comparison of different surgical techniques (without restriction by study design)—AR (restorative resection with or without temporary ileostomy) *versus* APE/TME or Hartmann’s (non-restorative resection with permanent/end stoma); QoL data provided by means of a validated tool; and published since last Cochrane review in 2012^16^.

Exclusion criteria were: lack of specific rectal cancer data (mixed data with colonic cancers but no subgroup data provided); unresectable disease or palliative surgery; local excision techniques; inclusion of surgery for inflammatory or benign growth; and not available in the English language^21^^,^^22^.

### Data analysis

Titles and abstracts of each article were screened before the whole paper being requested. Included papers underwent review. Patients who had undergone resection with bowel continuity restored were included in the AR group. The inclusion of patients with a temporary ileostomy vaired between papers, but this group included patients with a temporary ileostomy and those who had the ileostomy reversed. The APE group included all patients who had undergone non-restorative resection, including APE and HE.

Authors of articles with data presented in graphical form were contacted in an attempt to obtain numerical data; if there was no response from the corresponding author, the data were included in a summative analysis but not in the tables.

One reviewer extracted data into an electronic data collection sheet. A second independent reviewer checked this, with discrepancies resolved by consensus. Data extracted included: study-related data (first author, year of publication, journal, study design, duration of follow-up, outcomes measured, funding), patient characteristics (surgical technique, tumour site, neoadjuvant therapy) and outcome data (validated QoL measure results). Risk of bias was assessed using the Risk Of Bias In Non-Randomized Studies of Interventions (ROBINS-I) tool^23^; differences were resolved by consensus.

Narrative summary and qualitative analysis were planned, with comparison of the results between studies both during short- and long-term follow-up. Quantitative analysis through meta-analysis was considered inappropriate owing to clinical heterogeneity in interventions, the non-normal distribution of results, study design, and the variety of validated QoL outcome measures used.

### Outcome measures

The primary measure was difference in average global QoL. Other health-related items investigated were: gastrointestinal (GI) symptoms (stool frequency, flatulence, diarrhoea, and constipation), pain, and body image.

## Results

**Fig. 1:**
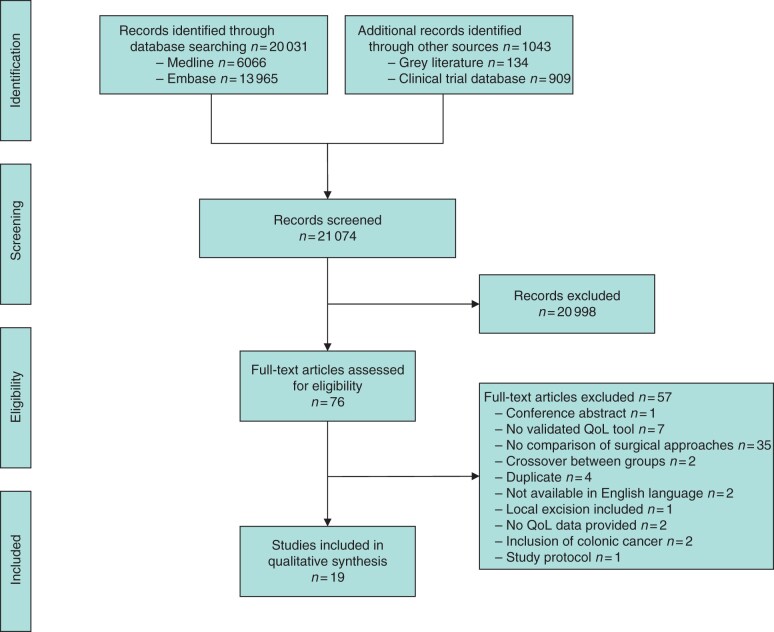
PRISMA diagram showing selection of articles for review QoL, quality of life.

Of 21 074 abstracts screened, 76 full papers were scrutinized and 19 included in the final review (*Fig. 1*)^24–42^. Nineteen studies met the inclusion criteria (*Table 1*) with a total of 6453 patients (range 43–1608). All articles described observational studies, although one study^37^ included patients from the National Surgical Adjuvant Breast and Bowel Project randomized trial (NSABP-R-04) from the USA. No patients were randomized between surgical techniques. Patient follow-up varied from 6 months to 5 years. Only four^30^^,^^34^^,^^35^^,^^37^ of the included studies provided preoperative QoL data and then followed patients up; two other papers^26^^,^^41^ provided serial QoL measurements, but not preoperative data. Thirteen studies provided only one measure of postoperative QoL, with no preoperative data. Fourteen studies used QLQ-CR30, eight used QLQ-CR29, and seven used QLQ-CR38 (*Table 1*). All studies compared outcomes for patients with rectal cancer; seven studies considered only rectal cancer within 4–6 cm of the anal verge, although it was not always stated how this was measured (*Table S1*). Surgical approaches and main findings are summarized in *Table 2*. Quantitative analysis was not completed because of the skewed data distribution, variety of QoL tools used, use of median (range), and the lack of standard deviation reporting^43^.

**Table 1:** Details of included studies

Reference	Year	Setting	Country	Research design	Duration of follow-up	No. of patients	QoL measure used
1. Mrak *et al.*^35^	2011	Single centre	Austria	Observational, prospectively maintained database	Minimum 3 years	59	EORTC QLQ-C30, EORTC QLQ CR29
2. How *et al.*^30^	2012	Single centre	UK	Observational, prospective	2 years	62	EORTC QLQ-C30, EORTC QLQ-CR38
3. Konanz *et al.*^33^	2013	Single centre, university- affiliated hospital	Germany	Observational, prospective database	Minimum 12 months	124	EORTC QLQ-C30, EORTC QLQ-CR38
4. Digennaro *et al.*^42^	2013	Multicentre	Italy	Observational, retrospective	Median 26.5 months (APE), 52.5 months (AR)	60	EORTC QLQ-C30, EORTC QLQ-CR38, Short Form 36
5. Arraras *et al.*^24^	2013	Single centre	Spain	Observational, prospective	Minimum 12 months	84	EORTC QLQ-C30, EORTC QLQ-CR29
6. Penchev *et al.*^36^	2014	Single centre, complex cancer centre	Bulgaria	Observational	Minimum 6 months	71	EORTC QLQ-CR38: sexual function
7. Russell *et al.*^37^	2015	Multicentre	USA	Observational, patients recruited to chemotherapy RCT	12 months	1608	FACT-C, EORTC QLQ-CR38
8. Feddern *et al.*^28^	2015	Population database	Denmark	Observational, cross-sectional survey	Minimum 4.2 years	1369	Brief Descriptive, Danish Pain Questionnaire (McKill)
9. Honda *et al.*^29^	2016	Single centre, cancer institute hospital	Japan	Observational, cross-sectional survey	Minimum 2 years	291	EORTC QLQ-C30, EORTC QLQ-CR29, modified FIQL
10. Monastyrska *et al.*^34^	2016	Single centre, oncology centre	Poland	Observational, prospective	6 months	100	EORTC QLQ-C30, EORTC QLQ-CR29
11. Klose *et al.*^31^	2017	Single centre, university- affiliated hospital	Germany	Observational, prospectively maintained database	58 months	143	EORTC QLQ-C30, EORTC QLQ-CR29
12. Wani *et al.*^40^	2017	Single centre, Kashmir	India	Observational, prospective	12 months	130	EORTC QLQ-C30, EORTC QLQ-CR29
13. Costa *et al.*^25^	2018	Single centre	Portugal	Observational, retrospective	21 months	43	International Index of Erectile Function
14. Koeter *et al.*^32^	2018	Population database	the Netherlands	Observational, longitudinal, prospective population-based survey	5.1 years	905	EORTC QLQ-C30, EORTC QLQ-CR38
15. Trenti *et al.*^39^	2018	Two centres	Spain	Observational, prospective	4.5 years	224	EORTC QLQ-C30, EORTC QLQ-CR29
16. Silva *et al.*^38^	2018	Single centre	Brazil	Observational, retrospective	3.84 years	125	EORTC QLQ-C30, EORTC QLQ-CR29
17. Du *et al.*^26^	2019	Single centre	China	Observational, retrospective	12 months after surgery	43	EORTC QLQ-C30, EORTC QLQ-CR38
18. Feddern *et al.*^27^	2019	Single centre	China	Observational, cross-sectional survey	Median 4.4 years	898	EORTC QLQ-C30
19. Ding *et al.*^41^	2020	Single centre	China	Observational, prospective	12 months	114	FIQL

Funding: 1. Not funded and no relationships to declare; 2. Pelican Cancer Foundation, Basingstoke; Colorectal Research Unit, North Hampshire Hospital, Basingstoke; 3. Not stated; 4. Not stated; 5. Grant 2443/2009 from Departamento de Salud del Gobierno de Navarra (Navarre Government Health Department), Spain; 6. Not stated; 7. NCI-U10-CA-12027, U10-CA-37377, U10-CA-69974, U10-CA-69651, and U10-CA-21661, by Roche; 8. Funding from private foundation of Daehnfeldt; 9. Japanese Foundation for Research and Promotion of Endoscopy; 10. Not stated; 11. Not stated; 12. Not stated; 13. Not stated; 14. Data collection funded by a VENI Grant (no. 451-10-041) from Netherlands Organization for Scientific Research awarded to F. Mols, together with a Medium Investment Grant from the Netherlands Organization for Scientific Research (NWO no. 480-08-009). D. Schoormans supported by a Social Psychology Fellowship from the Dutch Cancer Society (no. UVT2013-5893); 15. Not stated; 16. Not stated; 17. Grants from scientific research project of Gansu health industry (GSWST2013-03); 18. Danish Cancer Society; 19. Hunan Provincial Nature Science Foundation (2016JJ2106), Hunan Provincial Nature Science Foundation (2019JJ40510).

**Table 2:** Surgical intervention and study conclusions

Reference	Surgical intervention		Conclusions/recommendations	
	AR (restorative)	APE (non-restorative)	Global QoL	Symptoms
Mrak *et al.*^35^	Ultralow TME anterior resection and colonic J-pouch anastomosis	APE and end colostomy	QoL better after AR than APE in several respects	After AR, patients had better physical, role, cognitive, and social functioning with better body image. After APE, patients had significantly higher urinary frequency and were significantly more embarrassed by their condition
How *et al.*^30^	AR	APE and end colostomy	No difference in global QoL	There was more diarrhoea after AR and more pain at 2 years after operation. Better sexual and social functioning after AR
Konanz *et al.*^33^	ISR or LAR	APE and end colostomy	No difference in global QoL	Physical functioning significantly better after AR. Symptom scores for diarrhoea and constipation worse after AR
Digennaro *et al.*^42^	CAA (sewn and stapled)	APE and end colostomy	No difference in global QoL	APE group had worse sexual function, whereas most patients in AR group had faecal incontinence and sometimes obstructed defaecation, with an important impact on QoL
Arraras *et al.*^24^	LAR (with colorectal anastomosis or CAA)	APE and end colostomy	No difference in global QoL	Higher stool frequency and incontinence in AR group, but better emotional functioning
Penchev *et al.*^36^	AR	APE and end colostomy	Not compared	Sexual dysfunction worse in men after APE than AR
Russell *et al.*^37^	Sphincter-sparing surgery	Non-sphincter-sparing surgery	No difference in global QoL	AR group had better body image, male sexual enjoyment, and micturition symptoms. APR group had better GI tract symptoms and less weight loss. No difference in FACT-C
Feddern *et al.*^28^	AR (with TME or PME)	APE; Hartmann’s included but separate	Not compared	No association between pain intensity and type of surgery
Honda *et al.*^29^	Very low AR or ISR	APE and end colostomy	No difference in global QoL	Worse constipation, defaecation problems and anxiety in ISR group
Monastyrska *et al.*^34^	LAR (without stoma)	APE and end colostomy	No difference in global QoL	Physical, cognitive, and emotional functioning better in AR group
Klose *et al.*^31^	ISR	APE and end colostomy	No difference in global QoL	ISR group had better cognitive functioning and weight gain, and less nausea and vomiting, pain, dyspnoea, appetite loss, and embarassment. APE group had less diarrhoea, stool frequency, buttock pain, bloating, sore skin, and faecal incontinence
Wani *et al.*^40^	LAR	APE and end colostomy	No difference in global QoL	Nausea and vomiting worse in AR group, but urinary frequency, abdominal pain and embarassment worse in APE group
Costa *et al.*^25^	AR	APE and end colostomy	Not compared	APE and not AR is a risk factor for *de novo* ED
Koeter *et al.*^32^	LAR	APE and end colostomy	Not compared	No differences in physical activity between the two groups. Physical and role functioning seemed worse in APE group
Trenti *et al.*^39^	AR	APE and Hartmann’s	No difference in global QoL	Faecal incontinence worse in AR group and body image worse in APR group
Silva *et al.*^38^	Sphincter-saving surgery with closure of temporary ileostomy	APE and end colostomy	No difference in global QoL	APE group had significantly better functional and symptom scale scores
Du *et al.*^26^	AR with anal reconstruction	APE and end colostomy	No difference in global QoL	Emotional and social functioning better in AR group
Feddern *et al.*^27^	LAR	APE and end colostomy	Global health status worse in AR group	AR group had worse problems with diarrhoea and constipation
Ding *et al.*^41^	Ultralow AR (Dixon) and modified CAA (modified Parks)	Miles APE and end colostomy	Not compared	At 12 months, AR group had better scores in all four criteria of FIQL score

AR, anterior resection; APE, abdominoperineal excision; QoL, quality of life; TME, total mesorectal excision; ISR, intersphincteric resection; LAR, low anterior resection; CAA, coloanal anastomosis; GI, gastrointestinal; FACT-C, Functional Assessment of Cancer Therapy—Colorectal; PME, partial mesorectal excision; ED, Erectlie Dysfunction; FIQL, Faecal Incontinence Quality of Life Scale.

### Global quality of life

Only two studies included in the review identified a statistically significant difference in global QoL between restorative/AR and non-restorative/APE surgery. A single-centre study^35^ identified better QoL in patients who had undergone AR with colonic pouch formation at 13 months (*P* = 0.009), although no preoperative data were available. A population-based, cross-sectional study^27^ identified better global QoL in patients who had undergone APE (*P* = 0.026) at a median of 4.4 years after surgery, but again there were no preoperative QoL data. Extracted global QoL data are reported in *Table 3*. A difference of 10 on the EORTC 1–100 scale was used to compare the two surgical approaches; with the exception of one study^35^, no difference in global QoL between the two groups was noted^28^^,^^29^^,^^32^^,^^37^. Direction-of-effect analysis (based on whether a score at any time point after baseline favours AR or APR) found that four studies demonstrated better global QoL in patients who had undergone APE, four favoured AR, and three identified no difference. No link was identified when patients were separated by length of follow-up.

**Table 3:** Global quality-of-life measure using EORTC QLQ-CR30

	*n*	Surgical procedure	Global QoL score*					Direction of effect
			Baseline	0–5 months	6–11 months	1–2 years	> 2 years	
How *et al.*^30^†	62	APE	83 (33–100)			79 (33–100)	83 (17–100)	Trend favours APE but n.s.
		AR	79 (17–100)			71 (33–100)	75 (33–100)	
Mrak *et al.*^35^	59	APE					60.4 (20.1)	Favours AR (*P* < 0.050)‡
		AR					75.7 (20.1)	
Konanz *et al.*^33^	124	APE					59.2	Trend favours AR but n.s.
		AR					65.9	
		AR (ISR)					58.1	
Monastyrska *et al.*^34^	100	APE	51.7			60.5		Trend favours AR but n.s.
		AR	61.3			69		
Wani *et al.*^40^	130	APE				67.9(21.2)		Trend favours APE but n.s.
		AR				59.3(23.7)		
Arraras *et al.*^24^	84	APE				71.8(25.7)		Trend favours APE but n.s.
		AR				70.9(28.0)		
Du *et al.*^26^	43	APE		69.0(6.3)	69.4(6.4)	70.8(10.9)		Trend favours AR but n.s.
		AR		74.3(7.9)	75.4(8.9)	75.9(8.9)		
Trenti *et al.*^39^	224	APE					67.3 (21.4)	No difference
		AR					69.8 (24.6)	
		AR (CAA)					65.6 (23.4)	
Silva *et al.*^38^†	125	APE					75 (0–100)	No difference
		AR					75 (0–100)	
Digennaro *et al.*^42^†	60	APE					66.6 (50–100)	No difference
		AR					66.6 (16.7–100)	

*Values are mean(s.d.) unless indicated otherwise;

† values are median (range). The European Organization for Research and Treatment of Cancer (EORTC) QLQ-CR30 score has a range of 1–100, where 0 represents the best quality of life attainable. A score difference or change of 10 is claimed to be clinically important. Values are rounded to one decimal place. Articles with data represented graphically are not included in this table.

‡ *P* < 0.050 was considered statistically significant. QoL, quality of life; APE, abdominoperineal excision; AR, anterior resection; n.s., not statistically significant; ISR, intersphincteric resection; CAA, coloanal anastomosis.

### Pain

Extracted data for the pain domain of the validated QoL scores are presented in *Table 4*. Two studies identified a statistically significant reduction in pain after APE among patients who had follow-up longer than 2 years. Other studies with a clinically relevant difference on long-term follow-up also demonstrated reduced pain in the APE group. In long-term direction-of-effect analysis, five of the seven studies found reduced pain in the APE group. One study^40^ demonstrated increased pain in the APE group during follow-up of less than 12 months, but presented no preoperative data. No other studies identified a statistically significant or clinically relevant difference between the two groups. Direction-of-effect analysis showed that three studies identified no difference, although two with follow-up of less than 12 months favoured AR. Others^29^^,^^31^^,^^32^ with no numerical data available also showed no difference in postoperative pain. In one study^28^, multiple logistic regression showed that both AR (odds ratio (OR) 1.39, 95 per cent c.i. 1.01 to 1.90) and APE (OR 1.71, 1.19 to 2.44) were associated with chronic pelvic pain at a median of 4.2 years of compared with partial mesorectal excision.

**Table 4:** Validated measures of pain

	Surgical procedure	**Pain score** *					Direction of effect
		Baseline	0–5 months	6–11 months	1–2 years	> 2 years	
**QLQ-CR30: pain**							
How *et al.*^30^†	APE	0 (0–67)			0 (0–67)	0 (0–33)	Favours APE (*P* < 0.050)‡
	AR	0 (0–100)			17 (0–89)	33 (0–67)	
Mrak *et al.*^35^	APE					24.4	Trend favours AR but n.s.
	AR					17.5	
Konanz *et al.*^33^	APE					25.3	Trend favours AR but n.s.
	AR					17.5	
	AR (ISR)					22.7	
Monastyrska *et al.*^34^	APE	27			2		Trend favours APE but n.s.
	AR	23			9		
Wani *et al.*^40^	APE				18.5(21.9)		Trend favours APE but n.s.
	AR				26.3(29.9)		
Arraras *et al.*^24^	APE				23.8(26.1)		Trend favours AR but n.s.
	AR				17.9(25.5)		
Du *et al.*^26^	APE		10.3(4.3)	10.0(4.0)	9.1 (4.7)		No difference
	AR		10.1(4.7)	10.6(4.5)	9.8(5.8)		
Trenti *et al.*^39^	APE					12.1 (21.6)	Trend favours APE but n.s.
	AR					13.5 (20.9)	
	AR (CAA)					14.9 (21.1)	
Silva *et al.*^38^†	APE					0 (0–100)	Trend favours APE but n.s.
	AR					16.7 (0–100)	
**QLQ-CR29: abdominal pain**							
Wani *et al.*^40^	APE			32.1(26.4)			Trend favours AR but n.s.
	AR			9.3(22.2)			
Arraras *et al.*^24^	APE			12.7(24.7)			No difference
	AR			12.5(21.6)			
Silva *et al.*^38^†	APE					0 (0–66.7)	No difference
	AR					0 (0–100)	
**QLQ-CR29: buttock pain**							
Wani *et al.*^40^	APE			25.0(28.1)			Trend favours AR but n.s.
	AR			12.1(18.0)			
Arraras *et al.*^24^	APE			15.9(27.2)			No difference
	AR			16.7(24.6)			
Trenti *et al.*^39^	APE					11.7 (21.9)	Trend favours APE but n.s.
	AR					17.2 (27.5)	
	AR (CAA)					18.9 (27.2)	
Silva *et al.*^38^†	APE					0 (0–100)	Favours APE (*P* < 0.050)‡
	AR					0 (0–100)	

*Values are mean(s.d.) unless indicated otherwise;

† values are median (range). The European Organization for Research and Treatment of Cancer (EORTC) QLQ-CR30 and QLQ-CR29 scores have a range of 1–100, where 0 represents the lowest symptom burden. A score difference or change of 10 is claimed to be clinically important. Values are rounded to one decimal place. Articles with data represented graphically are not included in this table.

‡ *P* < 0.050 was considered statistically significant. APE, abdominoperineal excision; AR, anterior resection; n.s., not statistically significant; ISR, intersphincteric resection; CAA, coloanal anastomosis.

### Gastrointestinal symptoms

GI symptoms were measured using a variety of tools (EORTC QLQ-CR30, QLQ-CR30, QLQ-CR30) alongside the Faecal Incontinence Quality of Life Scale and Wexner scale after restorative surgery^44^^,^^45^ (*Table 5*). The use of specific tools to compare the two groups is challenging because of the difference in symptoms experienced by patients in the AR and APE groups. Long-term follow-up of greater than 2 years demonstrated favourable outcomes for APE over AR. In the domains of stool frequency, flatulence, GI symptoms, diarrhoea, and constipation, patients in the AR group had worse symptoms than those in the APE group. Only one study^34^, at 6 months but with no preoperative comparator, demonstrated better outcomes in the AR group; all the other studies either showed no difference or reported better outcomes in patients who had undergone APE.

**Table 5:** Validated scores for gastrointestinal symptoms

	Surgical procedure	Score*					Direction of effect
		Baseline	0–5 months	6–11 months	1–2 years	>2 years	
**QLQ-CR29: flatulence**							
Trenti *et al.*^39^	APE					31.5(26.8)	Favours APE (*P* < 0.050)‡
	AR					42.1(30.0)	
	AR (CAA)					56.7(30.5)	
Wani *et al.*^40^	APE			32.1(27.9)			No difference identified
	AR			33.7(28.3)			
Arraras *et al.*^24^	APE			33.3(26.5)			No difference identified
	AR			34.0(26.5)			
**QLQ-CR29: stool frequency**							
Trenti *et al.*^39^	APE					21.8(22.3)	Trend favours APE but n.s.
	AR					31.8(25.1)	
	AR (CAA)					40.0(26.5)	
Wani *et al.*^40^	APE			29.8(26.6)			No difference identified
	AR			28.4(24.3)			
Arraras *et al.*^24^	APE			14.3(18.5)			Favours APE (*P* < 0.050)‡
	AR			33.3(23.6)			
**QLQ-CR28: GI tract symptoms**							
Du *et al.*^26^	APE		20.1(8.4)	18.3(7.4)	15.1(5.5)		No difference identified
	AR		15.9(4.0)	15.5(3.6)	14.2(3.5)		
Russell *et al.*^37^	APE	21.4			15.2		No difference identified
	AR	16.8			18.9		
Konanz *et al.*^33^	APE					23.6	Trend favours APE but n.s.
	AR					32.5	
	AR (ISR)					37.8	
	APE			6.7			No difference identified
	AR			0			
**EORTC QLQ-CR30: nausea/vomiting**							
How *et al.*^30^†	APE	0 (0–33)			0 (0–33)	0 (0–33)	No difference identified
	AR	0 (0–33)			0 (0–33)	0 (0–33)	
Mrak *et al.*^35^	APE					6.7	No difference identified
	AR					3.8	
Konanz *et al.*^33^	APE					2.3	No difference identified
	AR					4.9	
	AR (ISR)					4.6	
Monastyrska *et al.*^34^	APE	11.3‡			13.7‡		Favours AR (*P* < 0.050)‡
	AR	4.7‡			7.4‡		
Wani *et al.*^40^	APE				8.1(18.1)		No difference identified
	AR				7.3(17.2)		
Arraras *et al.*^24^	APE				3.9(18.2)		No difference identified
	AR				5.2(17.5)		
Du *et al.*^26^	APE		7.7(5.6)	7.8(5.5)	5.8(5.9)		No difference identified
	AR		6.2(5.4)	6.1(4.4)	5.0(5.6)		
Trenti *et al.*^39^	APE					4.5 (15.3)	No difference identified
	AR					2.8 (8.4)	
	AR (CAA)					2.3 (7.4)	
Silva *et al.*^38^^†^	APE					(0–100)	No difference identified
	AR					(0–100)	
**EORTC QLQ-CR30: diarrhoea**							
How *et al.*^30^†	APE	33 (0–67)			0 (0–67)‡	0 (0–67)‡	Favours APE (*P* < 0.050)‡
	AR	0 (0–100)			33(0–100)‡	33 (0–67)‡	
Mrak *et al.*^30^	APE					16.7	Trend favours APE but n.s.
	AR					26.1	
Konanz *et al.*^33^	APE					16.7‡	Favours APE (*P* < 0.050)‡
	AR					34.1‡	
	AR (ISR)					45.5‡	
Monastyrska *et al.*^34^	APE	30.7			38.7‡		Favours AR (*P* < 0.050)‡
	AR	32			0.7‡		
Wani *et al.*^40^	APE				15.0(25.1)		No difference identified
	AR				16.7(32.1)		
Arraras *et al.*^24^	APE				11.1(19.2)		Trend favours APE but n.s.
	AR				21.4(27.3)		
Du *et al.*^26^	APE		9.8(8.0)	8.9(7.8)	8.7(7.8)		No difference identified
	AR		12.3(9.4)	11.8(7.3)	9.3(6.5)		
Trenti *et al.*^39^	APE					17.1(24.6)	Trend favours APE but n.s.
	AR					22.9(25.8)	
	AR (CAA)					27.8(27.8)	
Silva *et al.*^38^^†^	APE					0 (0-66.7)	No difference identified
	AR					0 (0-100)	
**EORTC QLQ-CR30: constipation**							
How *et al.*^30^†	APE	0 (0–100)			0 (0–67)‡	0 (0–33)	No difference identified
	AR	0 (0–67)			0 (0–100)‡	0 (0–67)	
Mrak *et al.*^35^	APE					14	No difference identified
	AR					21.6	
Konanz *et al.*^33^	APE					12‡	Favours APE (*P* < 0.050)‡
	AR					25.2‡	
	AR (ISR)					20.2‡	
Monastyrska *et al.*^34^	APE	36.67			16‡		Favours AR (*P* < 0.050)‡
	AR	23.3			0‡		
Wani *et al.*^40^	APE				15.5 (27.9)		No difference identified
	AR				15.4 (27.2)		
Arraras *et al.*^24^	APE				20.6 (24.7)		No difference identified
	AR				26.8 (33.9)		
Du *et al.*^26^	APE		13.9 (9.0)	13.7 (7.1)	12.1 (4.8)		No difference identified
	AR		15.8 (8.9)	14.5 (6.5)	13.0 (5.2)		
Trenti *et al.*^39^	APE					8.1 (19.2)‡	Favours APE (*P* < 0.050)‡
	AR					28.4 (32.1)‡	
	AR (CAA)					20.0 (24.1)‡	
Silva *et al.*^38^†	APE					0 (0–100)	No difference identified
	AR					0 (0–100)	

*Values are mean(s.d.) unless indicated otherwise;

† values are median (range). The European Organization for Research and Treatment of Cancer (EORTC) QLQ-CR30, QLQ-CR38, and QLQ-CR29 scores have a range of 1–100, where 0 represents the lowest symptom burden. A score difference or change of 10 is claimed to be clinically important. Values are rounded to one decimal place.

‡ *P* < 0.050 was considered statistically significant. APE, abdominoperineal excision; AR, anterior resection; CAA, coloanal anastomosis; n.s., not statistically significant; ISR, intersphincteric resection.

### Body image and sexual function

Five studies^26^^,^^32^^,^^35^^,^^37^^,^^39^ identified higher rates of negative body image in the APE group than the AR group, although preoperative data were not available. The other studies reported no difference; none reported better body image in the APE group (*Table 6*). Sexual function was worse in the APE group, but most studies that measured this did not have preoperative data (*Table 7*). Nine papers^24–26^^,^^30^^,^^33^^,^^35^^,^^37^^,^^38^^,^^40^ reported worse sexual functioning and/or interest in the APE group; five^29^^,^^31^^,^^32^^,^^34^^,^^39^ identified no difference between the two groups. One study^34^ identified worse functioning in the APE group, but this difference was present before operation and may reflect a difference in patient and tumour characteristics.

**Table 6:** Validated measures of body image

	Surgical procedure	Body image score*					Direction of effect
		Baseline	0–5 months	6–11 months	1–2 years	> 2 years	
Du *et al.*^26^	APE		75.1(11.4)	77.4(11.6)	79.9(9.4)		Favours AR (P < 0.050)‡
QLQ-CR38	AR		81.1(11.5)	84.4(8.9)	86.5(10.6)		
Mrak *et al.*^35^	APE					63.7(30.1)	Trend favours AR but n.s.
QLQ-CR29	AR					79.2(23.9)	
How *et al.*^30^†	APE	100 (50–100)			75 (25–100)	75 (25–100)	No difference identified
QLQ-CR38	AR	92 (33–100)			83 (0–100)	75 (33–100)	
Konanz *et al.*^33^	APE					62.4	Trend favours AR but n.s.
QLQ-CR38	AR					75.3	
	AR (ISR)					72.7	
Arraras *et al.*^24^	APE				92.1(11.7)		No difference identified
QLQ-CR29	AR				85.4(21.8)		
Wani *et al.*^40^	APE				84.1(15.0)		No difference identified
QLQ-CR29	AR				83.6(13.9)		
Trenti *et al.*^39^	APE					68.0(27.8)	Favours AR (P < 0.050)‡
QLQ-CR29	AR					81.9(26.2)	
	AR (CAA)					81.5(21.7)	
Silva *et al.*^38^†	APE					86.1 (0–100)	No difference identified
QLQ-CR29	AR					88.9 (0–100)	
					

*Values are mean(s.d.) unless indicated otherwise;

† values are median (range). The European Organization for Research and Treatment of Cancer (EORTC) QLQ-CR38 and QLQ-CR29 scores have a range of 1–100, where 0 represents the lowest symptom burden. A score difference or change of 10 is claimed to be clinically important. Values are rounded to one decimal place. Articles with data represented graphically are not included in this table.

‡ *P* < 0.050 was considered statistically significant. APE, abdominoperineal excision; AR, anterior resection; n.s., not statistically significant; ISR, intersphincteric resection; CAA, coloanal anastomosis.

**Table 7:** Validated measures of sexual function

	Surgical procedure	Baseline	0–5 months	6–11 months	1–2 years	> 2 years	Direction of effect
**Sexual interest**							
Mrak *et al.*^35^ QLQ-CR29	APE					23.8(25.2) F 33.6(33.2) M	Trend in women favours AR but n.s. Favours AR in men (*P* < 0.050)‡
	AR					40.7(27.8) F 51.2(35.7) M	
Wani *et al.*^40^ QLQ-CR29	APE				41.0(20.0) F 45.2(24.8) M		Trend favours AR but n.s
	AR				56.6(34.8) F 64.4(36.2) M		
Trenti *et al.*^39^ QLQ-CR29	APE				11.1(28.0) F 40.5(31.5) M		Trend favours AR but n.s.
	AR				20.0(25.8) F 42.3(33.2) M		
	AR (CAA)				14.3(17.8) F 46.0(30.7) M		
Silva *et al.*^38^† QLQ-CR29	APE				66.7 (0–100) F 33.3 (0–100) M		No difference identified
	AR				66.7 (33.3–100) F 33.3 (0–100) M		
**Sexual enjoyment**							
How *et al.*^30^†	APE	16.5 (0–100)			17 (0–100)	33 (0–67)	Trend favours APE at 2 years but n.s.
QLQ-CR38	AR	50 (0–100)			67 (0–100)	16 (0–67)	
Konanz *et al.*^33^	APE					56.4	Favours AR (*P* < 0.050, ISR *versus* APE)‡
QLQ-CR38	AR					53.7	
	AR (ISR)					75.9	
Penchev *et al.*^36^	APE			27.7(31.2)			Trend favours APE but n.s.
QLQ-CR38	AR			18.9(15.1)			
Du *et al.*^26^	APE		41.7(16.0)	46.7(16.0)	51.7(14.2)		Favours AR (*P* < 0.050)‡
QLQ-CR38	AR		52.1(12.3)	56.7(12.1)	60.1(11.6)		
**Sexual functioning**							
How *et al.*^30^†	APE	0(0–83)			0(0–67)	0(0–33)	Favours AR (*P* < 0.050)‡
QLQ-CR38	AR	33(0–83)			33(0–67)	33(0–100)	
Konanz *et al.*^33^	APE					21.7	Favours AR (*P* < 0.050, ISR *versus* APE)‡
QLQ-CR38	AR					31.7	
	AR (ISR)					44.4	
Arraras *et al.*^24^	APE						No difference identified
QLQ-CR29	AR				3.5(10.5) F 31.2(35.4) M	4.8(12.6) F 36.1(38.8) M	
Penchev *et al.*^36^	APE			9.6(15.9)			No difference identified
QLQ-CR38	AR			10.36(16.3)			
Du *et al.*^26^	APE		38.8(17.2)	42.1(17.9)	46.6(14.5)		Favours AR (*P* < 0.050, ISR *versus* APE)‡
QLQ-CR38	AR		46.6(13.3)	52.0(13.1)	56.1(14.2)		

*Values are mean(s.d.) unless indicated otherwise;

† values are median (range). The European Organization for Research and Treatment of Cancer (EORTC) QLQ-CR38 and QLQ-CR29 scores have a range of 1–100, where 0 represents the lowest symptom burden. A score difference or change of 10 is claimed to be clinically important. Values are rounded to one decimal place. Articles with data represented graphically are not included in this table.

‡ *P* < 0.050 was considered statistically significant. APE, abdominoperineal excision; AR, anterior resection; n.s., not statistically significant; ISR, intersphincteric resection.

### Risk of bias

**Fig. 2:**
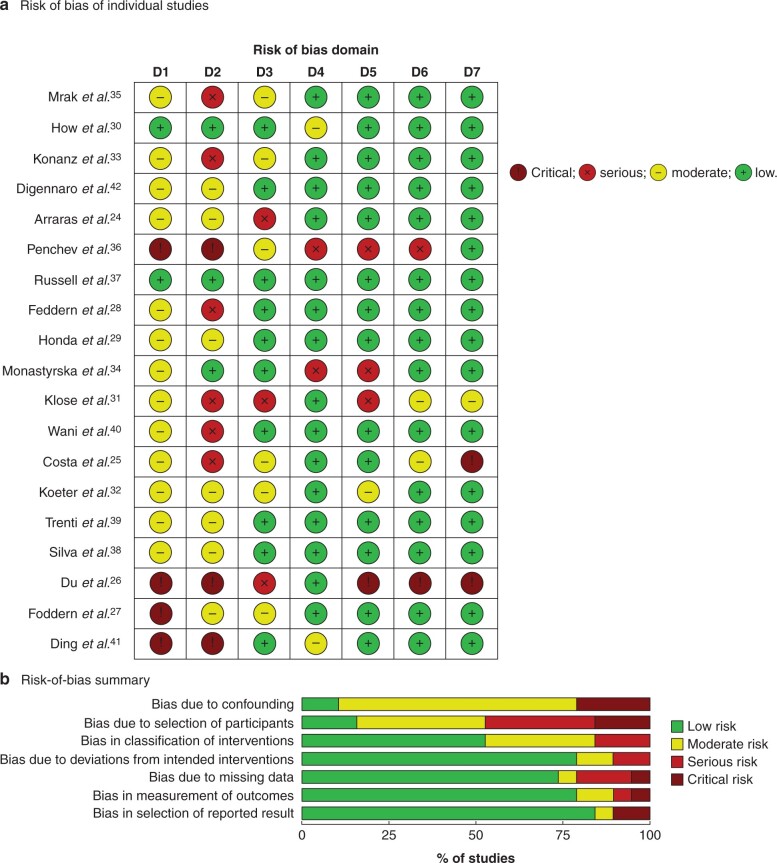
Risk-of-bias assessment **a** Risk of bias in individual trials. D1, bias due to confounding; D2, bias due to selection of participants; D3, bias in classification of interventions; D4, bias due to deviations from intended interventions; D5, bias due to missing data; D6, bias in measurement of outcomes; D7, bias in selection of reported result. **b** Risk-of-bias summary.

The ROBINS-I risk-of-bias assessment was completed for all studies. All bar one had at least a low/moderate risk of bias. Twelve had a serious risk of bias in at least one domain and five had a critical risk of bias in at least one domain (*Fig. 2*). Two had a low risk of bias in more than six domains, both of which found no difference in global QoL. The reason for high risk of bias varied between studies. Recurring themes included the non-reporting of patient characteristics including co-morbidities, different disease profiles, different preoperative chemoradiotherapy regimens between the groups, and variation in questionnaire completion.

## Discussion

Overall, there was no improvement in global QoL across the studies; restorative surgery was not found to improve QoL compared with a permanent stoma. However, some caution is required in interpretation of the research because published data were at significant risk of bias and no high-quality papers existed to allow accurate analysis of the difference in QoL. Different symptom profiles were identified; when studies reported differences of statistical value, patients who underwent APE had worse body image and those who underwent AR had worse GI symptoms.

The ROBINS-I assessment of all the studies reflected this high risk of bias and demonstrated the paucity of good-quality studies aimed at assessing this clinically relevant question. This should be considered when assessing the conclusions of the review. The risk of bias was contributed to by the paucity of preoperative QoL data, the use of single-point QoL scores, and failure to control for the location of the rectal cancer. The distance of the cancer from the anal verge is paramount in deciding surgical and neoadjuvant treatments, and therefore has a significant impact on postoperative outcomes. Two studies included in the review had a low risk of bias in 6 or more domains. One report found no difference in global QoL, but reported better cognitive and social functioning with fewer symptoms of pain, diarrhoea, sleep disturbance, and constipation in patients who had undergone APE. Another documented no difference in global QoL between the two surgical techniques; however, no raw data were included in the publication and it was not therefore included in any Tables. The same authors identified worse sexual function and micturition symptoms in patients who had undergone APE, but they had better GI symptom profiles.

Postoperative differences in QoL measures may have been present before surgery and therefore cannot be explained simply by differences in surgical technique. The importance of ensuring that the disease profile is matched should be highlighted; controlling for tumour height and neoadjuvant therapy is important as these have an impact on patient QoL. This may explain the results of many of the studies included in this review. The lack of preoperative QoL measures to identify any possible differences being caused by variation in surgical indication rather than surgical technique introduces significant potential bias into most studies included in the review. It is therefore challenging to identify whether disease location, preoperative differences in QoL or operative approach is the reason for a difference in postoperative QoL. Variation in duration of follow-up may reflect different aspects of the patient journey. Long-term follow-up may miss significant short-term variation in QoL and will miss patients with short postoperative survival; however, longer follow-up allows good assessment of function. The variability of follow-up and grouping of patients across these time brackets may mean a mixed picture is provided across the included studies. Many studies included patients with a large range of follow-up times and it is therefore difficult to draw specific conclusions regarding changes in QoL over time.

The high search volume reflects the large amount of work being done regarding QoL outcomes after rectal cancer surgery. Many articles were excluded, as they did not offer a comparison between restorative and non-restorative rectal procedures. The comparison of observational data between single-arm studies further increases the risk of bias, which was therefore avoided by exclusion of papers with no direct comparison between groups. Selection bias in observational studies in the review will have been increased further owing to patient selection for different techniques. The use of non-validated tools for postoperative QoL was also commonplace and did not allow accurate and reliable conclusions to be drawn from the data. An example is the use of QoL questions that had not been assessed to demonstrate validity, reliability or to ensure that they provided a true reflection of the patient experience. There was variation between studies, with heterogeneity in results identified. The variety of inclusion criteria, differing levels of neoadjuvant therapy, differences in follow-up time, and range of surgical techniques is likely to be reflected in the differences in results.

Most studies did not specify location of the rectal cancer as an inclusion criterion; higher rectal tumours suitable for AR and not for APE will create selection bias because of a lower risk of developing low anterior resection syndrome, and produce more favourable outcomes in the AR group. The cohort of patients who underwent AR in the included studies often excluded those who had not undergone ileostomy reversal. The non-closure rate of defunctioning ileostomies 18 months after AR was 25.1–30 per cent^46^^,^^47^ and these patients are considered to have permanent loop ileostomies. The exclusion of these patients, therefore, is not reflective of clinical practice. These patients may have had their QoL improved by having an end colostomy at initial operation rather than living with a loop ileostomy and its attendant challenges of dietary restrictions, skin irritation, and renal impairment, although this was not addressed in the present analysis. Some studies excluded patients who had a postoperative anastomotic leak. Such leaks have a significant impact on long-term QoL and therefore introduce significant bias into the relevant studies. The exclusion of patients with a permanent ileostomy, patients at higher risk of low anterior resection syndrome, and those who had an anastomotic leak may reflect favourably on patients who have undergone AR and not reflect clinical practice. This, therefore, does not allow surgeons to provide patients with accurate information.

The results of this systematic review are in keeping with previously published work. The Cochrane review^16^ published in 2012 found equipoise in QoL outcomes and was also unable to recommend AR over APE. A previous meta-analysis^48^ from 2007 also identified no difference in QoL outcomes after AR *versus* APE for rectal cancer. The present systematic review supports these findings in studies that have been published since the Cochrane review in 2012. Data published since this date should also allow for the introduction of enhanced recovery after surgery protocols, the use of preoperative MRI, and should not include the laparoscopic learning curve. These subsequent studies may therefore be more relevant to current practice. Another study^49^ published since completion of the search also identified no difference in global QoL; however, although patients who had undergone APE had lower preoperative QoL, this was not accounted for in the conclusion that patients should undergo restorative surgery for low rectal cancer.

These results should be discussed with patients as part of shared decision-making and consenting for operative management of rectal cancer, although this review cannot recommend one surgical approach over another for improved QoL. Future studies should record detailed clinical factors alongside properly validated preoperative QoL measures used for patients undergoing both surgical approaches. These studies should include only patients with low rectal cancer, as previously defined in the literature^50^^,^^51^, to allow direct comparison between techniques and reduce selection bias. Patients should be followed up adequately with the same QoL measures used after surgery, and both short- and long-term data collected. The use of the collaborative research model may provide a framework for this work. The Colostomy Impact Score (CIS) and the Low Anterior Resection Score (LARS) both now have validated convergence on the EORTC QLQ-C30, and may therefore be useful in allowing a direct comparison between the two surgical techniques^52–54^. The impact of ileostomy on patients’ QoL should be considered, and may not be assessed accurately by the CIS and LARS. Additional work is required to understand the process by which surgeons decide which operations to offer the individual patient.

*Disclosure*. The authors declare no conflict of interest.

## Supplementary material

Supplementary material is available at *BJS Open* online.

## Supplementary Material

zrab101_Supplementary_Data

## References

[1] Mercury Group. Diagnostic accuracy of preoperative magnetic resonance imaging in predicting curative resection of rectal cancer: prospective observational study. BMJ 2006;333:779. 16984925 10.1136/bmj.38937.646400.55 PMC1602032

[2] Plummer JM , Leake PA, Albert MR. Recent advances in the management of rectal cancer: no surgery, minimal surgery or minimally invasive surgery. World J Gastrointest Surg 2017;9:139–148. 28690773 10.4240/wjgs.v9.i6.139 PMC5483413

[3] Heald RJ , Smedh RK, Kald A, Sexton R, Moran BJ. Abdominoperineal excision of the rectum–an endangered operation. Norman Nigro Lectureship. Dis Colon Rectum 1997;40:747–751. 9221846 10.1007/BF02055425

[4] Morris E , Quirke P, Thomas JD, Fairley L, Cottier B, Forman D. Unacceptable variation in abdominoperineal excision rates for rectal cancer: time to intervene? Gut 2008;57:1690–1697. 18535029 10.1136/gut.2007.137877

[5] Jayne D , Pigazzi A, Marshall H, Croft J, Corrigan N, Copeland J et al Effect of robotic-assisted *vs* conventional laparoscopic surgery on risk of conversion to open laparotomy among patients undergoing resection for rectal cancer: the ROLARR randomized clinical trial. JAMA 2017;318:1569–1580. 29067426 10.1001/jama.2017.7219 PMC5818805

[6] Liao G , Li YB, Zhao Z, Li X, Deng H, Li G. Robotic-assisted surgery *versus* open surgery in the treatment of rectal cancer: the current evidence. Sci Rep 2016;6:26981. 27228906 10.1038/srep26981 PMC4882598

[7] Acuna SA , Chesney TR, Ramjist JK, Shah PS, Kennedy ED, Baxter NN. Laparoscopic *versus* open resection for rectal cancer: a noninferiority meta-analysis of quality of surgical resection outcomes. Ann Surg 2019;269:849–855. 30339624 10.1097/SLA.0000000000003072

[8] Bonjer HJ , Deijen CL, Abis GA, Cuesta MA, van der Pas MHGM, de Lange-de Klerk ESM et al; COLOR II Study Group. A randomized trial of laparoscopic *versus* open surgery for rectal cancer. N Engl J Med 2015;372:1324–1332. 25830422 10.1056/NEJMoa1414882

[9] Ma B , Gao P, Song Y, Zhang C, Zhang C, Wang L et al Transanal total mesorectal excision (taTME) for rectal cancer: a systematic review and meta-analysis of oncological and perioperative outcomes compared with laparoscopic total mesorectal excision. BMC Cancer 2016;16:380. 27377924 10.1186/s12885-016-2428-5 PMC4932707

[10] Simillis C , Hompes R, Penna M, Rasheed S, Tekkis PP. A systematic review of transanal total mesorectal excision: is this the future of rectal cancer surgery? Colorectal Dis 2016;18:19–36. 26466751 10.1111/codi.13151

[11] Thaysen HV , Jess P, Laurberg S, Groenvold M. Validation of the Danish version of the disease specific instrument EORTC QLQ-CR38 to assess health-related quality of life in patients with colorectal cancer. Health Qual Life Outcomes 2012;10:150. 23241096 10.1186/1477-7525-10-150 PMC3541093

[12] Whistance RN , Conroy T, Chie W, Costantini A, Sezer O, Koller M et al; European Organisation for the Research and Treatment of Cancer Quality of Life Group. Clinical and psychometric validation of the EORTC QLQ-CR29 questionnaire module to assess health-related quality of life in patients with colorectal cancer. Eur J Cancer 2009;45:3017–3026. 19765978 10.1016/j.ejca.2009.08.014

[13] Crott R , Briggs A. Mapping the QLQ-C30 quality of life cancer questionnaire to EQ-5D patient preferences. Eur J Health Econ 2010;11:427–434. 20473703 10.1007/s10198-010-0233-7

[14] Ramsey SD , Berry K, Moinpour C, Giedzinska A, Andersen MR. Quality of life in long term survivors of colorectal cancer. Am J Gastroenterol 2002;97:1228–1234. 12017152 10.1111/j.1572-0241.2002.05694.x

[15] Antonescu I , Carli F, Mayo NE, Feldman L. Validation of the SF-36 as a measure of postoperative recovery after colorectal surgery. Surg Endosc 2014;28:3168–3178. 24879142 10.1007/s00464-014-3577-8

[16] Pachler J , Wille‐Jørgensen P. Quality of life after rectal resection for cancer, with or without permanent colostomy. Cochrane Database Syst Rev 2012; (12)CD004323. 10.1002/14651858.CD004323.pub4 PMC7197443 23235607

[17] NHS Improvement. *Living with and Beyond Cancer: Taking Action to Improve Outcomes*. https://assets.publishing.service.gov.uk/government/uploads/system/uploads/attachment_data/file/181054/9333-TSO-2900664-NCSI_Report_FINAL.pdf (accessed 15 February 2021).

[18] General Medical Council Council. Good Medical Practice. https://www.gmc-uk.org/-/media/documents/good-medical-practice–-english-20200128_pdf-51527435.pdf?la=en&hash=DA1263358CCA88F298785FE2BD7610EB4EE9A530 (accessed 15 February 2021).

[19] Moher D , Liberati A, Tetzlaff J, Altman DG; PRISMA Group. Preferred reporting items for systematic reviews and meta-analyses: the PRISMA statement. PLoS Med 2009;6:e1000097. 19621072 10.1371/journal.pmed.1000097 PMC2707599

[20] Shea BJ , Reeves BC, Wells G, Thuku M, Hamel C, Moran J et al AMSTAR 2: a critical appraisal tool for systematic reviews that include randomised or non-randomised studies of healthcare interventions, or both. BMJ 2017;358:j4008. 28935701 10.1136/bmj.j4008 PMC5833365

[21] Morrison A , Polisena J, Husereau D, Moulton K, Clark M, Fiander M et al The effect of English-language restriction on systematic review-based meta-analyses: a systematic review of empirical studies. Int J Technol Assess Health Care 2012;28:138–144. 22559755 10.1017/S0266462312000086

[22] Jüni P , Holenstein F, Sterne J, Bartlett C, Egger M. Direction and impact of language bias in meta-analyses of controlled trials: empirical study. Int J Epidemiol 2002;31:115–123. 11914306 10.1093/ije/31.1.115

[23] Sterne JA , Hernán MA, Reeves BC, Savović J, Berkman ND, Viswanathan M et al ROBINS-I: a tool for assessing risk of bias in non-randomised studies of interventions. BMJ 2016;355:i4919. 27733354 10.1136/bmj.i4919 PMC5062054

[24] Arraras JI , Suarez J, Arias-de-la-Vega F, Vera R, Ibanez B, Asin G et al Quality of life assessment by applying EORTC questionnaires to rectal cancer patients after surgery and neoadjuvant and adjuvant treatment. Rev Esp Enferm Dig 2013;105:255–261. 23971656 10.4321/s1130-01082013000500003

[25] Costa P , Cardoso JM, Louro H, Dias J, Costa L, Rodrigues R et al Impact on sexual function of surgical treatment in rectal cancer. Int Braz J Urol 2018;44:141–149. 29219281 10.1590/S1677-5538.IBJU.2017.0318 PMC5815544

[26] Du P , Wang SY, Zheng PF, Mao J, Hu H, Cheng ZB. Comparison of overall survival and quality of life between patients undergoing anal reconstruction and patients undergoing traditional lower abdominal stoma after radical resection. Clin Transl Oncol 2019;21:1390–1397. 31006088 10.1007/s12094-019-02106-x

[27] Feddern ML , Emmertsen KJ, Laurberg S. Quality of life with or without sphincter preservation for rectal cancer. Colorectal Dis 2019;21:1051–1057. 31074098 10.1111/codi.14684

[28] Feddern ML , Jensen TS, Laurberg S. Chronic pain in the pelvic area or lower extremities after rectal cancer treatment and its impact on quality of life: a population-based cross-sectional study. Pain 2015;156:1765–1771 26010459 10.1097/j.pain.0000000000000237

[29] Honda M , Akiyoshi T, Noma H, Ogura A, Nagasaki T, Konishi T et al Patient-centered outcomes to decide treatment strategy for patients with low rectal cancer. J Surg Oncol 2016;114:630–636 27761895 10.1002/jso.24376

[30] How P , Stelzner S, Branagan G, Bundy K, Chandrakumaran K, Heald RJ et al Comparative quality of life in patients following abdominoperineal excision and low anterior resection for low rectal cancer. Dis Colon Rectum 2012;55:400–406 22426263 10.1097/DCR.0b013e3182444fd1

[31] Klose J , Tarantino I, Kulu Y, Bruckner T, Trefz S, Schmidt T et al Sphincter-preserving surgery for low rectal cancer: do we overshoot the mark? J Gastrointest Surg 2017;21:885–891 27981492 10.1007/s11605-016-3339-0

[32] Koeter T , Bonhof CS, Schoormans D, Martijnse IS, Langenhoff BS, Zimmerman DDE et al Long-term outcomes after surgery involving the pelvic floor in rectal cancer: physical activity, quality of life, and health status. J Gastrointest Surg 2019;23:808–817 30374817 10.1007/s11605-018-4014-4

[33] Konanz J , Herrle F, Weiss C, Post S, Kienle P. Quality of life of patients after low anterior, intersphincteric, and abdominoperineal resection for rectal cancer—a matched-pair analysis. Int J Colorectal Dis 2013;28:679–688 23571868 10.1007/s00384-013-1683-z

[34] Monastyrska E , Hagner W, Jankowski M, Głowacka I, Zegarska B, Zegarski W. Prospective assessment of the quality of life in patients treated surgically for rectal cancer with lower anterior resection and abdominoperineal resection. Eur J Surg Oncol 2016;42:1647–1653 27514720 10.1016/j.ejso.2016.07.007

[35] Mrak K , Jagoditsch M, Eberl T, Klingler A, Tschmelitsch J. Long-term quality of life in pouch patients compared with stoma patients following rectal cancer surgery. Colorectal Dis 2011;13:e403–e410 21812896 10.1111/j.1463-1318.2011.02740.x

[36] Penchev D , Masliankov S, Todorov G. Assessment of the sexual function after rectal cancer surgery. Khirurgiia (Sofiia) 2014;14–20 26152060

[37] Russell MM , Ganz PA, Lopa S, Yothers G, Ko CY, Arora A et al Comparative effectiveness of sphincter-sparing surgery *versus* abdominoperineal resection in rectal cancer: patient-reported outcomes in National Surgical Adjuvant Breast and Bowel Project randomized trial R-04. Ann Surg 2015;261:144–148 24670844 10.1097/SLA.0000000000000594 PMC4379325

[38] Silva M , Junior SA, de Aguiar Pastore J, Santos EMM, de Oliveira Ferreira F, Spencer R et al Late assessment of quality of life in patients with rectal carcinoma: comparison between sphincter preservation and definitive colostomy. Int J Colorectal Dis 2018;33:1039–1045 29675592 10.1007/s00384-018-3044-4 PMC6060835

[39] Trenti L , Galvez A, Biondo S, Solis A, Vallribera-Valls F, Espin-Basany E et al Quality of life and anterior resection syndrome after surgery for mid to low rectal cancer: a cross-sectional study. Eur J Surg Oncol 2018;44:1031–1039 29665980 10.1016/j.ejso.2018.03.025

[40] Wani RA , Bhat IU, Parray FQ, Chowdri NA. Quality of life after ‘Total Mesorectal Excision (TME)’ for rectal carcinoma: a study from a tertiary care hospital in Northern India. Indian J Surg Oncol 2017;8:499–505 29203980 10.1007/s13193-017-0698-2 PMC5705521

[41] Ding H , Li J, Chen Y, Yang Z, Peng Z, Liao X. Anal function and quality of life analysis after laparoscopic modified Parks for ultra-low rectal cancer patients. World J Surg Oncol 2020;18:28 32013992 10.1186/s12957-020-1801-7 PMC6998312

[42] Digennaro R , Tondo M, Cuccia F, Giannini I, Pezzolla F, Rinaldi M et al Coloanal anastomosis or abdominoperineal resection for very low rectal cancer: what will benefit, the surgeon’s pride or the patient’s quality of life? Int J Colorectal Dis 2013;28:949–957 23274737 10.1007/s00384-012-1629-x

[43] Higgins J , Thomas J, Chandler J, Cumpston M, Li T, Page MJ, Welch VA (eds). *Cochrane Handbook for Systematic Reviews of Interventions Version 6.1* (*Updated September* 2020*).* http://www.training.cochrane.org/handbook (accessed 15 February 2021).

[44] Rockwood TH , Church JM, Fleshman JW, Kane RL, Mavrantonis C, Thorson AG et al Fecal incontinence quality of life scale: quality of life instrument for patients with fecal incontinence. Dis Colon Rectum 2000;43:9–16 10813117 10.1007/BF02237236

[45] Jorge JM , Wexner SD. Etiology and management of fecal incontinence. Dis Colon Rectum 1993;36:77–97 8416784 10.1007/BF02050307

[46] Dukes’ Club Research Collaborative. Factors impacting time to ileostomy closure after anterior resection: the UK closure of ileostomy timing cohort study (CLOSE-IT). Colorectal Dis 2021;23:1109–1119 33452859 10.1111/codi.15531

[47] National Gastrointestinal Audit Project Board. National Bowel Cancer *Audit* 2020. https://www.nboca.org.uk/content/uploads/2020/12/NBOCA-2020-Annual-Report.pdf. (accessed 15 February 2021).

[48] Cornish JA , Tilney HS, Heriot AG, Lavery IC, Fazio VW, Tekkis P. A meta-analysis of quality of life for abdominoperineal excision of rectum versus anterior resection for rectal cancer. Ann Surg Oncol 2007;14:2056–2068 17431723 10.1245/s10434-007-9402-z

[49] Kang SB , Cho JR, Jeong SY, Oh JH, Ahn S, Choi S et al Quality of life after sphincter preservation surgery or abdominoperineal resection for low rectal cancer (ASPIRE): a long-term prospective, multicentre, cohort study. Lancet Reg Health West Pac 2021;6:100087 34327411 10.1016/j.lanwpc.2020.100087 PMC8315365

[50] D’Souza N , de Neree Tot Babberich MPM, d'Hoore A, Tiret E, Xynos E, Beets-Tan RGH et al Definition of the rectum: an international, expert-based Delphi consensus. Ann Surg 2019;270:955–959 30973385 10.1097/SLA.0000000000003251

[51] Moran BJ , Holm T, Brannagan G, Chave H, Quirke P, West N et al The English National Low Rectal Cancer Development Programme: key messages and future perspectives. Colorectal Dis 2014;16:173–178 24267315 10.1111/codi.12501

[52] Smart N. Stomas: time for a closer look. Colorectal Dis 2017;19:1049 29193580 10.1111/codi.13911

[53] Emmertsen KJ , Laurberg S. Low anterior resection syndrome score: development and validation of a symptom-based scoring system for bowel dysfunction after low anterior resection for rectal cancer. Ann Surg 2012;255:922–928 22504191 10.1097/SLA.0b013e31824f1c21

[54] Thyø A , Emmertsen KJ, Pinkney TD, Christensen P, Laurberg S. The colostomy impact score: development and validation of a patient reported outcome measure for rectal cancer patients with a permanent colostomy. A population-based study. Colorectal Dis 2017;19:O25–O33. 27883253 10.1111/codi.13566

