# Supplementary material for: Quality of life in restorative versus non-restorative resections for rectal cancer: systematic review
Source: BJS Open. 2022 Jan 18;5(6):zrab101. doi: 10.1093/bjsopen/zrab101 (PMC8765336; doi:10.1093/bjsopen/zrab101)
Supplement: zrab101_Supplementary_Data [file zrab101_supplementary_data.zip › Supplementary_material.docx]

**Supplementary material**

Search strategy

| **Search term – Medline & EMBASE** |
| --- |
| 1. surg* or operat* or intervention* or procedur* or resect* |
| 2. (colo* or rect*) adj2 (canc* or carc* or neopla* or adenocar* or tumo* or malig*) |
| 3. (functio* adj2 outco*) or (patien* adj2 outcom*) or (quality adj2 li*) or ((late or long term) adj2 morbidit*) |
| 4. 1. and 2. and 3. |

**British Library Thesis repository & Grey Literature search engine & Clinicaltrials.gov search**

“Rectal Cancer”
